# Geriatric oral health neglect: calculus on the attached gingiva: a case report

**DOI:** 10.1097/MS9.0000000000002230

**Published:** 2024-06-04

**Authors:** Udohchukwu Promise Okereke, Chimeremeze Offurum, Victor Anezi Eze, Chidubem Peter Okpechukwu, Wisdom Chisom Ifedibar, Ezi Abigail Akaji

**Affiliations:** a Faculty of Dentistry, College of Medicine, University of Nigeria; b YOHAN Research Institute, Nsukka; c Irrua Specialist Hospital, Irrua; d Department of Preventive Dentistry, Faculty of Dentistry, University of Nigeria, Enugu Campus, Nsukka

**Keywords:** calculus, geriatric, gingiva, oral health, primary healthcare, public health

## Abstract

**Introduction and importance:**

Aging exerts various effects on and causes changes to the oral tissues. It is often difficult to distinguish between what is caused by the physiological process of aging itself and what is caused by the individual’s lifestyle or diseases. The elderly face peculiar health challenges and require special dental care. It is therefore vital that greater attention be given to the oral health needs of this rapidly population. In this article, the authors present the case of an elderly female patient with a rare case of calculus on the attached gingiva.

**Case presentation:**

A 90-year-old retired teacher with a history of hypertension, presented with a small, painless, grayish-brown growth on her attached gingiva, noticed by her daughter-in-law. Examination revealed signs of gingivitis and significant dental issues including missing teeth, fractures, and calculus buildup. Diagnosis of chronic generalized marginal gingivitis and calculus deposition was made, and scaling and polishing were performed. Perforation of the attached gingiva was observed during scaling thus exposing the root, which facilitated the calculus accumulation. Post-procedure care included medication and oral hygiene instructions. Follow-up after 1 week showed satisfactory healing, but subsequent assessment at 3 months revealed plaque accumulation, with the patient declining further treatment.

**Clinical discussion:**

The patient shows relative neglect in oral healthcare given the subpar oral health features she exhibited and decline of further treatment options presented to her. This is common in the population as some abnormal oral presentations and features are perceived as normal in the population, which can be attributed to poor knowledge of oral health practices, which affects the illness seeking behaviour of individual.

**Conclusion:**

Calculus deposition in the oral cavity requires a hard surface for formation, and proper oral hygiene practices are essential to mitigate its adverse effects, particularly among the elderly who may require additional attention due to their unique physiological changes.

## Introduction

Highlights

- The elderly population are underserved in terms of their oral healthcare.
- The aging population shows neglect in their oral healthcare.
- Calculus requires a hard material for its formation and deposition in the mouth.
- There is a need for special attention to the oral health needs of the elderly population in our society.
- A multisectoral approach is needed in providing wholesome care to the elderly and aging population.

Dental calculus consists of mineralized bacterial plaque, typically concealed by a tightly adherent non-mineralized plaque during its formation^1–5^. It contains both organic and inorganic components, with the organic constituting ~15–20% of its dry weight. This matrix includes amino acids, peptides, glycoproteins, proteins, carbohydrates, and lipids^2,4^. The inorganic constituents, primarily calcium and phosphorus, along with carbonate, sodium, magnesium, and fluoride, closely resemble those found in enamel, bone, dentin, and cementum, even exhibiting similar crystal forms^3,4^.

Aging is a physiological and heterogeneous process that can predispose individuals to pathologies. Globally, there is an increase in the proportion of people living into their old age due to various reasons such as improvement in living standards, advances in healthcare and widespread availability of medicines. These factors interact to cause an increase in life expectancy, with people generally living longer today than they previously did in the past. However, aging comes with several physiological changes that can pose a challenge to the health and wellbeing of the individual^5^.

Aging exerts various effects on and causes changes to the oral tissues. It is often difficult to distinguish between what is caused by the physiological process of aging itself and what is caused by the individual’s lifestyle or diseases. However, with increasing age, the oral cavity undergo some changes with the teeth demonstrating wearing off of the enamel, chipping and fracture lines, and a darker colour^6^, the pulp chambers are progressively reduced in size^7^ and the alveolar bones undergo atrophy with age, due to periodontitis, tooth loss and edentulousness, and the pressure of dentures on the bone^8^. Reduced salivation and xerostomia is also common in the elderly. This is due to both systemic diseases like diabetes, and the use of medications, many of which cause hyposalivation as a side effect^9^.

As people age, they become more prone to acute and chronic diseases, as well as life-threatening conditions^6^. Cardiovascular diseases, cancer, diabetes, digestive disorders, joint pains, and infections become more frequent in the elderly. But also, poor oral health, manifesting as tooth loss and edentulism, and severe periodontal diseases is common in this group^6^.

Previous studies have shown that poor oral hygiene in elderly people, both institutionalized and those living at home, is a significant health problem^10^. Several factors in this group interact and result in this poor oral health status. These factors range from poor personal oral hygiene practices to systemic diseases and conditions, and even to socioeconomic factors of the elderly. The presence of systemic diseases and disabilities in the elderly negatively affects their oral health. Some of the drugs taken predispose them to caries and periodontal disease. Neurodegenerative diseases and reduced motor ability in the elderly may compromise oral hygiene practices and hinder access to dental services^5,11^. Lack of financial resources for oral health and decreased routine utilization of dental health services lead to late recognition of disease, therefore escalating the severity of existing disease conditions, and possible tooth loss^6,11^. Some socioeconomic variables such as education level, income, social support network and psychosocial status have been reported to be associated with the oral health status of the elderly^5^.

Previous studies have shown that the elderly often have poor oral hygiene status and high plaque and calculus deposits. In another study in Saudi Arabia by Aljoharah A. Al-Sinaidi among elderly Saudis in a nursing home in Riyadh found that the most prevalent oral condition among the subjects was calculus (48.2%), and only 8.4% of the subjects had healthy periodontium^12^. Another study among the elderly in Tonga, Cameroon by Yotat Michele and colleagues found that their oral hygiene practices were far below standard and visible plaque was found on the surface of the teeth of 41.4% of the participants. Their periodontal status was generally poor as the patients presented with high calculus deposits and more than a third had periodontal pockets of 4–6 mm, likely due to improper brushing methods^11^.

The elderly face peculiar health challenges and require special dental care. It is therefore vital that greater attention be given to the oral health needs of this rapidly population. In this article, we present the case of an elderly female patient with a rare case of calculus on the attached gingiva, which has never been reported before The findings of this study will shed more light on the oral health of geriatric population, which has been supported by relevant literature.

This case report has been reported in line with the SCARE Criteria^13^.

The FDI tooth notation system has been used in charting all teeth mentioned within this study^14^.

### Case report

A 90-year-old woman and a known hypertensive and peptic ulcer patient who presented on account of small growth on her attached gingiva. It was noticed by her daughter-in-law who called to her attention. Her vital signs were normal (BP - 120/80 mmHg, Temp - 36.6°c, Pulse - 76 bpm). Patient is under management for rheumatoid arthritis and has had a positive history of transient ischaemic attack, which has been managed. She is been cared for by her daughters in-law and grandchildren.

**Figure 1:**
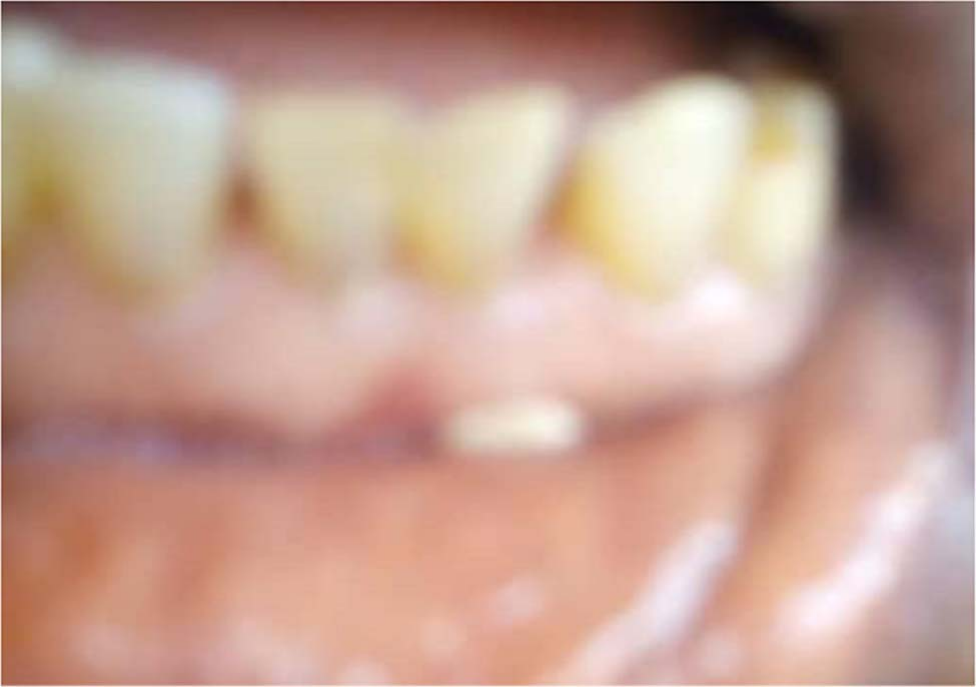
Calculus deposition on the attached gingiva about 1 cm below the lower left lateral incisor.

**Figure 2:**
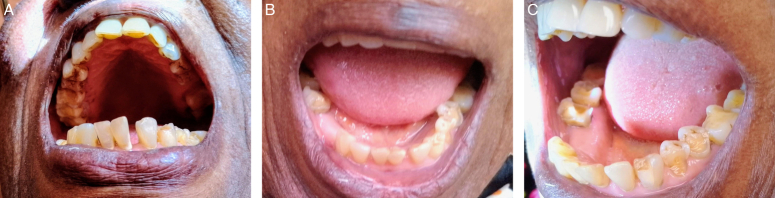
(A) Oral cavity showing intraoral tissues. Notice the plaque deposits on the lower anterior teeth.*All figure were taken on 3-month follow-up. (B) Intraoral tissues on the lower jaw. Notice the missing teeth and the other abnormalities. *All Figure were taken on 3-month follow-up. (C) Patient’s intraoral status. See the attrition, fracture and stains seen in the patient’s mouth. *All Figure were taken on 3-month follow-up.

On general examination, there were no notable features. The extraoral examinations were clinically normal. On intraoral examination, there was a hard, painless, and grayish-brown mass found at about 1 cm below the lower left lateral incisor on the attached gingiva (Fig. 1), measuring about 2×2×3 mm. The soft tissues appeared clinically normal except the gingiva, which showed signs of gingivitis evidenced by loss of stippling, loss of knife-edge appearance and erythematous colour change of the gum. There was presence of debris, calculus and stains but no halitosis was perceived; however, the oral hygiene index was fair with an Oral Hygiene Index (OHI-S) of 2. The hard tissues showed that the 26, 44 and 45 were missing (FDI notation) while all the posteriors showed significant attrition. The posterior teeth, 34, 35, 46 and 47 were fractured, and there were stains, debris and calculi present (Fig. 2A, B, C). Also, the lower incisors and right canine teeth exhibited grade 2 mobility.

After examination, diagnosis of Chronic Generalized Marginal Gingivitis and calculus on attached gingiva was made, and scaling and polishing was carried out on the patient by the corresponding author, a graduate dentist, with Oral Hygiene instructions given immediately after the procedure. During the scaling, the calculus buildup on the attached gingiva was removed and it was seen that the layer it attached on had been perforated thus exposing the root of that tooth, therefore giving the needed environment for calculus deposition and accumulation.

The mild bleeding was controlled and the procedure completed with the patient placed on warm water mouth gargle for 3 days, Zinc phosphate tablet, 200 mg daily for 3 days, Vitamin C tablet 500 mg BD for 5 days and Paracetamol tablet, 500 mg BD for 3 days.

On follow-up one week later, healing was spontaneous, and recovery was satisfactory. Patient went about her normal life activities. Consequently, 3-month follow-up showed complete coverage of the area by regenerated gingival tissues; however, it revealed plaque accumulation on the buccolingual aspects of the lower anterior teeth. Patient declined further treatment interventions.

## Discussion

The formation of dental calculus has been a relatively understudied concept for years. However, it is generally believed that the process initiates with the deposition of the pellicle, an organic layer coating tooth surfaces, facilitated by saliva or gingival crevice fluid. This pellicle swiftly becomes colonized by microorganisms, predominantly bacteria, deriving nutrients primarily from oral fluids’ proteins, glycoproteins, amino acids, and peptides, rather than ingested food items passing through the oral cavity^2^. Mineralization can commence within a few days of microbial colonization of the pellicle, although the precise onset of this process can vary significantly. Throughout mineralization, calcium phosphate crystals are deposited in the organic plaque matrix. This matrix comprises not only the microorganisms but also amorphous material derived from them and from the oral fluids^3–5^.

Calculus deposition can be supragingival or subgingival when it is deposited above or below the free gingiva, respectively. For supragingival calculus, the greatest facilitator has been noted to be saliva, while gingival crevice fluid is the driver for the subgingival calculus deposition. There is a direct correlation between the degree of production of crevicular fluid and the degree of periodontal inflammation, hence greater amounts of subgingival calculus might be expected in sites with moderate to severe inflammation^15^. Also, Supragingival calculus deposits are usually seen more on the surfaces of teeth close to the openings of the ducts of the major salivary glands, such as the lingual aspects of the mandibular anterior teeth and the buccal aspects of the maxillary molars, whereas subgingival calculus deposits have no predilection to those sites^15^.

Meanwhile, on our patient, the calculus deposited on the attached gingiva as seen in Fig. 1 which has never been recorded before and as such a novel presentation thus questioning the belief that plaque and calculus requires a dental hard material for its formation^16,17^ However, after the dislodgement of the deposit it was seen that the deposit was on an exposed root. This therefore, further consolidates the understanding that calculus forms on hard materials through the simultaneous effects of other mediating factors such as microbial activities, interplay between the organic and inorganic components in the oral cavity, health of the periodontium and other factors due to individual’s lifestyle. The root exposure might have been due to an unknown trauma that was not well investigated and treated but became unnoticed and relatively painless on the deposition of the calculus.

The soft and hard tissue features of the patient can be said to be as expected in that the presence of missing teeth is common in the elderly population as well as the poor periodontal health exhibited. Some studies have shown that with increasing age, the mean number of remaining teeth is fewer than 20 and a person’s lifetime exposure to various risk factors such as poor oral hygiene, tobacco smoking, trauma^16–19^, and general medical conditions contributes to an elevated prevalence of periodontal problems in the elderly. Periodontal disease, a result of these cumulative risk factors, heightens the likelihood of developing root caries and chewing difficulties, ultimately potentially leading to tooth loss^16–20^.

Furthermore, given the patient’s age, age changes are expected to have set in; however, that does not fully account for the status of her oral health rather her lifestyle and oral health practices such as toothbrushing frequency and techniques, diet and nutrition has more profound effect on her oral health outcomes as evidenced by the accumulation of plaques and calculus on her teeth post scaling treatment after 3 months. A study in Germany by Anna-Luisa Klotz and colleagues, which compared the oral hygiene status of people aged 50 and 70 years old found that the mean Plaque Index triples from 0.4 in 50 year olds to 1.2 in 70 year olds.11.

In all, the patient shows relative neglect in oral healthcare given the subpar oral health features she exhibited and decline of further treatment options presented to her. This is common in the population as some abnormal oral presentations and features are perceived as normal in the population, which can be attributed to poor knowledge of oral health practices, which affects the illness seeking behaviour of individual^20–22^.

In addressing oral health issues in the elderly population, it’s imperative to integrate oral healthcare into primary healthcare (PHC) activities, incorporating the Basic Package of Oral Care (BPOC). Additionally, a review of National Health Insurance Scheme (NHIS) policies regarding oral health is necessary to ensure adequate coverage for elderly individuals. Advocacy efforts are essential to change policymakers’ perceptions about the importance of oral health in relation to overall health among the elderly. This advocacy should target individuals at local, state, and federal levels, emphasizing the significance of oral health in maintaining quality of life for older adults.

Furthermore, it’s crucial to alter the perceptions of other healthcare professionals regarding oral health education, collaboration, and dental referrals in the context of elderly care. Training programs and collaborative initiatives should highlight the specific oral health needs of the elderly population and promote interdisciplinary approaches to care.

Strengthening oral health education methods tailored to the social determinants of health in the elderly population is vital. These educational efforts should consider factors such as access to care, mobility issues, and socioeconomic status to ensure that oral health interventions are effective and impactful in improving the oral health outcomes of older adults^20–22^.

Gerondontology should be wholly incorporated into the dental curriculum in the undergraduate, postgraduate, and continuing education levels of the dental profession as some developed countries have done in order to equip the dental practitioner with the necessary knowledge and skills to attend to the elderly patient; this will greatly Carter for the elderly population and as well reduce the currently high burden of oral diseases among the population^22–25^.

## Conclusion

Calculus deposition requires a hard material in the oral cavity for its formation irrespective of the site of formation. Proper oral health practices are essential to mitigate the deleterious effects of calculus deposition in the oral cavity among both the elderly and general population. However, the elderly population may require more attention given their peculiarities, which is not limited to the physiological age changes they experience. To achieve this improved level of care of oral and general healthcare among this population, a constellation of approaches that is not limited to increased oral health education, improved oral health policies integration of oral healthcare into the PHC and improved gerondontology education within dental training, needs to be undertaken to effectively give a wholistic healthcare to the elderly population.

## Ethical approval

Ethical approval for this study was provided by the College of Medicine Research Ethics Committee (COMREC) of the College of Medicine, University of Nigeria Enugu Campus on 30th December, 2023 with the protocol number 0152/12/2023.

## Consent

Written informed consent was obtained from the patient for publication of this case report and accompanying images. A copy of the written consent is available for review by the Editor-in-Chief of this journal on request.

## Source of funding

Not applicable.

## Author contribution

O.P.U. saw and managed the patient, O.C. and E.V.A. reviewed the patient. E.A.A., O.C.P. and I.W.C. wrote the first draft of the paper. All authors reviewed the manuscript critically and approved the final draft.

## Conflicts of interest disclosure

The author declares no conflict of interest.

## Research registration unique identifying number (UIN)

Not applicable.

## Guarantor

Okereke Promise Udohchukwu.

## Data availability statement

Not applicable.

## Provenance and peer review

Not commissioned, externally peer-reviewed.
